# Asymmetric Soil Warming under Global Climate Change

**DOI:** 10.3390/ijerph16091504

**Published:** 2019-04-28

**Authors:** Hui Zhang, Binhui Liu, Daowei Zhou, Zhengfang Wu, Ting Wang

**Affiliations:** 1 School of Geographical Sciences, Northeast Normal University, Changchun 130024, China; zhangh636@nenu.edu.cn; 2 College of Forestry, The Northeast Forestry University, Harbin 150040, China; lbinhui@yahoo.com; 3 Northeast Institute of Geography and Agroecology, Chinese Academy of Sciences, Changchun 130102, China; wangting@iga.ac.cn

**Keywords:** asymmetric, soil temperature, SDTR, increase, decrease

## Abstract

Daily surface soil temperature data from 360 weather stations in China during 1962–2011 were retrieved and analyzed. The data revealed two aspects of asymmetric soil warming. Firstly, there was asymmetry between day and night in terms of increases in soil temperature. The daily maximum surface soil temperature ($ST_{\max}$) and daily minimum surface soil temperature ($ST_{\min}$) increased at rates of 0.031 and 0.055 °C/year over the 50-year interval, respectively. As a consequence of the more rapid increases in $ST_{\min}$, the soil diurnal temperature range (SDTR) decreased at most stations (average rate of –0.025 °C/year), with the most profound decrease in winter (–0.08 °C/year). The solar duration (SD) was positively related to SDTR and is regarded as the key underlying cause of the decreasing SDTR. Secondly, there was asymmetry between the soil and air in the temperature increase. The differences between soil and air temperature ($T_{D}$) were highest in summer (2.76 °C) and smallest in winter (1.55 °C), which decreased by 0.3 °C over the study interval, this meant agricultural practice plans based on air temperature alone may be severely limited. The difference between soil temperature and air temperature reduces at night. This would facilitate the wintering of perennials in areas near the zero-contour line.

## 1. Introduction

A large proportion of the globe has experienced climate change since the 20th century. Using the global mean surface air temperature, it has been determined that the reported increases in mean daily temperatures are due, at least partially, to differential changes in the maximum and minimum temperatures [1]. On most continents, the diurnal temperature range, defined as the difference between daily maximum and minimum temperatures, proved to be decreasing, this is due to the obvious increase in night temperatures, and less distinct or absent increases in day temperatures [1]. Numerous changes in climatic and environmental conditions as well as local land use can affect DTR (diurnal range of air temperature) [2,3,4,5,6]. In addition, there is a growing consensus that the asymmetrical diurnal warming of air affects various ecological processes [7,8,9,10,11]. In China, DTR has significantly decreased on both regional and national scales over the past several decades. On a regional basis, the magnitude of the decrease was greater in northern China and smaller in southern China [5,6,12,13,14]. 

Soil temperatures may play important roles in agriculture production and human activity. Global warming has caused changes in soil temperatures. IPCC5 (Fifth Assessment Report of the Intergovernmental Panel on Climate Change) reported that the global mean trends in ground temperature significantly increased for all datasets over multi-decadal periods [15]. The analysis of the data from 292 stations in the United States showed that the soil temperature (depth of 10 cm) increased by 0.32 °C over 10 years [16]. Zhang et al. found that the soil temperature in Canada in the 20th century experienced significant warming, with the complex response process of soil temperature to air temperature and precipitation changes having an obvious impact on climate change [17]. In China, scholars have also paid more attention to the changes in soil temperature. A published paper reported that the annual surface soil temperature increased by 1.90 °C (national level) during 1961–2011, with the highest changes occurring in the northern area and during winter. The warming occurred to a depth of up to 3.20 m [18].

However, the warming trends of surface soil temperatures and its change characteristics, not only in China but elsewhere, have not been categorized in great detail over prolonged intervals. The diurnal change in surface soil temperature, which is similar to the change in air temperature, is directly affected by various above-ground environmental conditions. Furthermore, most soil processes, including the decomposition and formation of soil organic matter, are sensitive to diurnal temperature changes [9,18]. Consequently, the asymmetry of soil temperature (i.e., SDTR: diurnal range of soil temperature) affects regional atmospheric circulation and numerous soil processes [19,20,21]. Therefore, rigorous analyses of soil surface temperature are of great importance despite the difficulty in accessing reliable and complete long-term data.

The overall objective of this study was to determine trends in soil temperatures from 1962 to 2011 using data from 360 national standard meteorological stations across China. Our specific objectives were to determine: (1) Spatiotemporal changes in soil temperature ($ST_{\max}$, $ST_{\min}$ and SDTR); (2) linkages between SDTR and other climate variables in order to elucidate the underlying causes of changes in SDTR; and (3) relationships between soil temperature and air temperature, $T_{D}$.

## 2. Materials and Methods 

### 2.1. Data Source and Collation

Data were obtained from the China Meteorological Administration and Provincial Meteorological Administration, encompassing 759 national standard meteorological stations across China. Unfortunately, complete long-term monitoring soil temperature data were not available for every station. The quality of the soil temperature data was poorer and it was less complete compared to the air temperature data, especially in the 1950s. This was due to instrument malfunctions and changes in administration. Hence, we started our study from the 1960s.

The raw data used in this paper included $ST_{mean}$, $ST_{\max}$, $ST_{\min}$, and SDTR at 0 cm from 1962 to 2011 (Table 1). The stations also reported the monthly means of $AT_{\mathrm{mean}}$, $AT_{\max}$, $AT_{\min}$, and DTR; the monthly means of SD, h, and V; and the monthly accumulation of p during the study period (Table 1). 

First, we wanted to establish a qualified dataset. For all variables related to soil temperature and for any given month to be included, there had to be at least 20 days of daily records for that month. This criterion reduced the number of stations to 360. For any month, if the calculated average temperature differed from the 50-year average ($\bar{A}$) (1962–2011) for that month by at least three times the standard deviation of $\bar{A}$, that value was treated as an outlier and was replaced by the average of the three nearest stations for that month and year. However, the three nearest stations need to be as evenly distributed as possible. After this quality check, for any daily or monthly mean, the annual ($A_{i},i=1,2,\cdots 50$) values of soil temperature were used for the following assessments. 

Secondly, the monthly air temperatures and other meteorological variables across China had their quality directly tested by the China Meteorological Administration due to their high quality. Hence, we just used the monthly air temperatures and other meteorological variables data in the same 360 stations in order to be consistent with soil temperature data. We also defined $T_{D}$ as the differences between the mean surface soil temperature and mean air temperature. In essence, $T_{D}=ST_{mean}-AT_{mean}$. 

Finally, to assess the spatial patterns of temperature changes, China was divided into 50 regions in a 5° × 5° grid. The data from individual stations contained within each region were averaged to calculate the regional and national mean values. 

### 2.2. Data Calculation and Analyses

The trends for the change in every variable ($ST_{mean}$, $ST_{\max}$, $ST_{\min}$, SDTR, $AT_{\mathrm{mean}}$, $AT_{\max}$, $AT_{\min}$, DTR, SD, h, V, p and $T_{D}$) across 360 stations were estimated by the Mann–Kendall test and a simple linear regression using monthly mean values. For each season, namely winter (Dec–Feb), spring (Mar–May), summer (Jun–Aug), and autumn (Sept–Nov), the seasonal mean soil temperature was calculated first from those three months before seasonal trends were obtained. Annual trends were similarly calculated, with seasonal trends based on the monthly average soil temperatures.

Correlation coefficients (SAS statistical software, liner, SAS Institute Inc., Cary, NC, USA) were calculated between the soil temperature variables and other climate variables, including SD, p, h, and V in order to estimate relationships between them (Table 2). Furthermore, we also calculated partial correlation coefficients (SAS statistical software, liner, SAS Institute Inc., Cary, NC, USA) between the variables and SDTR to eliminate the effects of any interaction among variables on SDTR. 

## 3. Results and Discussion

### 3.1. Annual and Seasonal Trends

**Figure 1:**
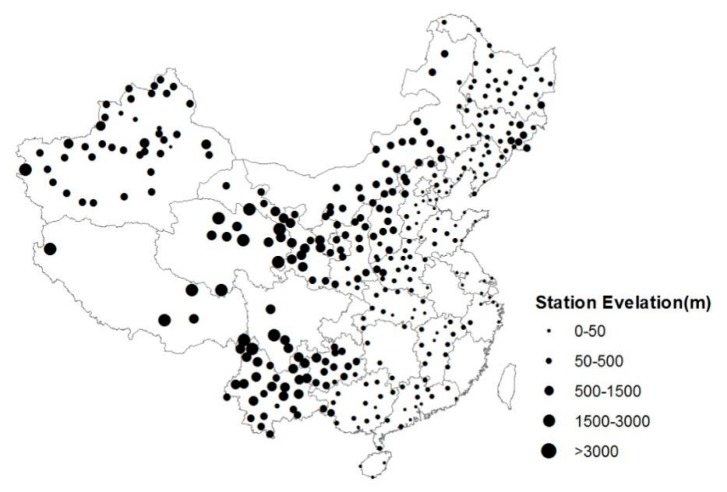
Geographical distribution of the 360 weather stations used in this study.

**Figure 2:**
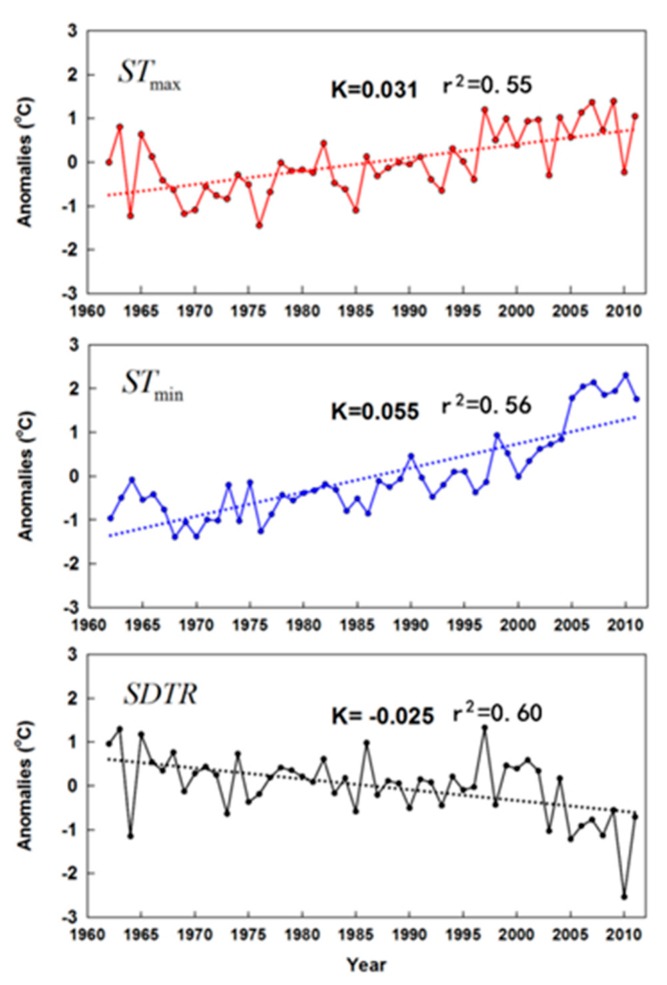
Linear trends and anomalies from annual means of $ST_{\max}$, $ST_{\min}$, and SDTR in China during 1962–2011. Dashed lines are trend lines. K represents the slope (i.e., rate of change (°C/year)); all the slopes of trends were significant (*p* < 0.05). Anomaly is the difference between the temperature of one year (corresponding to the X-axis) and the average temperature of 50 years.

Taking China as a whole (Figure 1), the changes in the mean $ST_{\max}$, $ST_{\min}$, and SDTR were separately calculated from 1962 to 2011 based on the edited dataset. There were significant increases in $ST_{\max}$ (0.031 °C/year) and $ST_{\min}$ (0.055 °C/year) across China during the study period (Table 2, Figure 2). The obvious difference in the soil warming between day and night resulted in a narrowing of SDTR at a rate of 0.025 °C/year, which is consistent with air temperature changes in previous studies [5,6,22]. $AT_{\max}$ and $AT_{\min}$ increased by 0.221 and 0.378 °C/decade, respectively, while DTR decreased at a rate of 0.157 °C/decade nationally [6]. Seasonal trends in soil temperature variables were generally consistent with annual trends. Changes in $ST_{\max}$ were highest in spring (0.048 °C/year) and smallest in winter (0.017 °C/year; not significant) during the study period (Table 2). In addition, the increase in $ST_{\min}$ was highest in winter (0.097 °C/year) and smallest in summer (0.034 °C/year; Table 2). For winter, $ST_{\max}$ increased slightly, whereas $ST_{\min}$ increased greatly. This is inconsistent with a previous report that the increases in $AT_{\max}$ and $AT_{\min}$ were both highest in winter on a nationwide scale [6]. Therefore, the modest increases in $ST_{\max}$(constrained by precipitation), combined with substantial increases in $ST_{\min}$, collectively resulted in substantial decreases in SDTR (–0.080 °C/year). These decreases in SDTR primarily occurred in winter and there were non-significant decreases in SDTR during summer and autumn and a non-significant increase during spring. Therefore, the annual decrease in SDTR was primarily attributed to the large decrease in winter. This dominant influence of winter on DTR is consistent with previous studies [5,6]. 

### 3.2. Spatial Patterns

**Figure 3:**
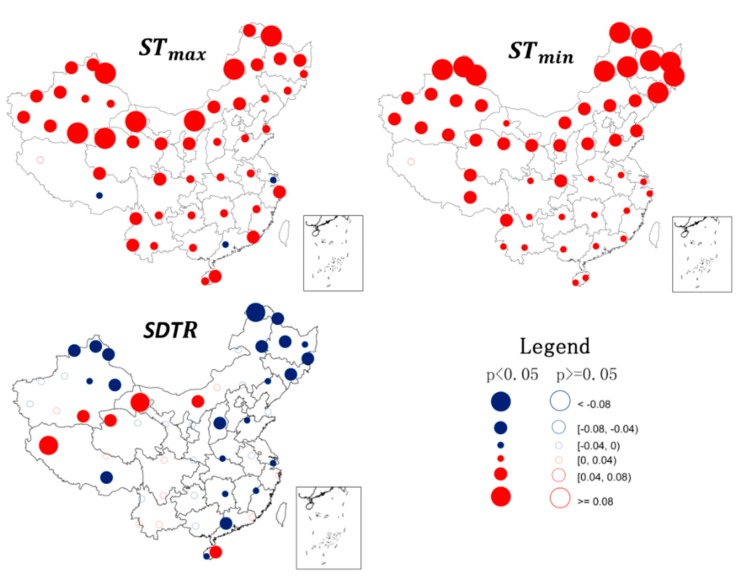
Spatial patterns of changes in soil surface temperatures ($ST_{\max}$, $ST_{\min}$, and SDTR) during 1962–2011 (°C/year) at 360 observational stations in China. The color of the circles indicates the magnitude of change. Solid circles with point indicate significance level of *p* < 0.05, whereas hollow circles are *p* > 0.05. Maps in this figure were created by ArcGIS 9.3 (ESRI, 2008).

The annual trends of $ST_{\max}$, $ST_{\min}$, and STR for each of the 360 stations were calculated separately. It was evident that soil warming occurred across most of China, particularly in the northeast (Figure 3). This is consistent with the previous results of changes in air temperature in China [5,6], with only Shanghai Province, Guangdong Province, and southeast Tibet having a cooling trend in $ST_{\max}$.

Based on a transect from north to south, the warming trend in $ST_{\min}$ changed from high to low (Figure 3). For $ST_{\max}$, this increase reduced both from north to south and from west to east. For some stations, there was actually a cooling trend for $ST_{\max}$. Most of these stations were located in northern China, where irrigation has been implemented to enhance agriculture. Presumably, the evapotranspiration during summer contributed to the obvious decrease in $ST_{\max}$ in this area [23]. In general, either $ST_{\min}$ rose at a faster pace than $ST_{\max}$ or $ST_{\min}$ kept increasing, whereas $ST_{\max}$ decreased. As a consequence, SDTR decreased for most stations, although a few stations had pronounced increases in SDTR.

**Figure 4:**
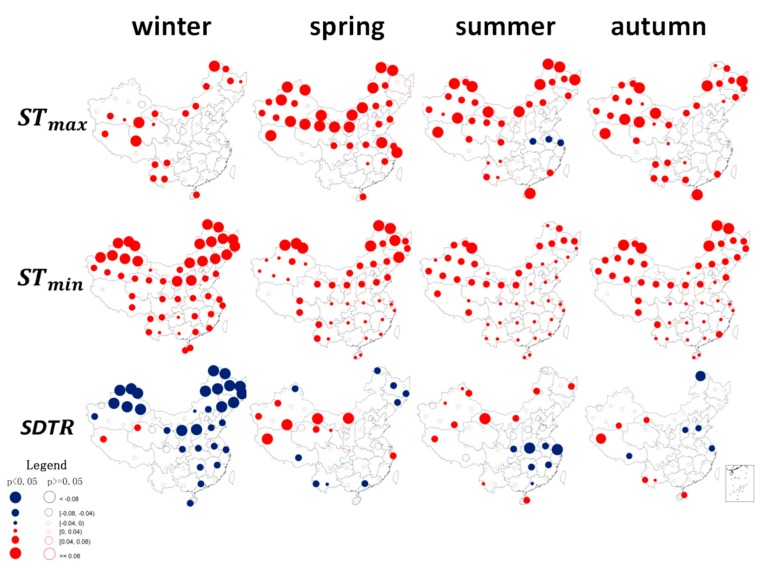
Spatial and seasonal patterns of changes in soil surface temperatures ($ST_{\max}$, $ST_{\min}$, and SDTR) during 1962–2011 (°C/year) at 360 observational stations in China. The color of the circles indicates the magnitude of change. Solid circles with point indicate significance level of *p* < 0.05, whereas hollow circles are *p* > 0.05. Maps in this figure were created by ArcGIS 9.3 (ESRI, 2008).

The seasonal and annual trends were similar, with large changes in $ST_{\min}$ in the northern regions and small changes in the southern regions in each season. Furthermore, the largest changes occurred in winter. Some of these differences were attributed to the summer monsoon, which accounts for the majority of the year’s precipitation and contributes to the south being much wetter than the north (Figure 4). The stations experiencing the highest warming were located in western China, most of Inner Mongolia, a portion of the northeast and Yunnan Province. In the rainy summer months, $ST_{\max}$ decreased across large areas of central China (Figure 4), which substantially decreased the SDTR. In the fall, the reduced trend in $ST_{\max}$ mainly occurred in Hebei Province. Based on these regional disparities in SDTR across China, we inferred that the changes in $ST_{\max}$ were the primary cause.

### 3.3. Possible Factors Contributing to SDTR Change

**Figure 5:**
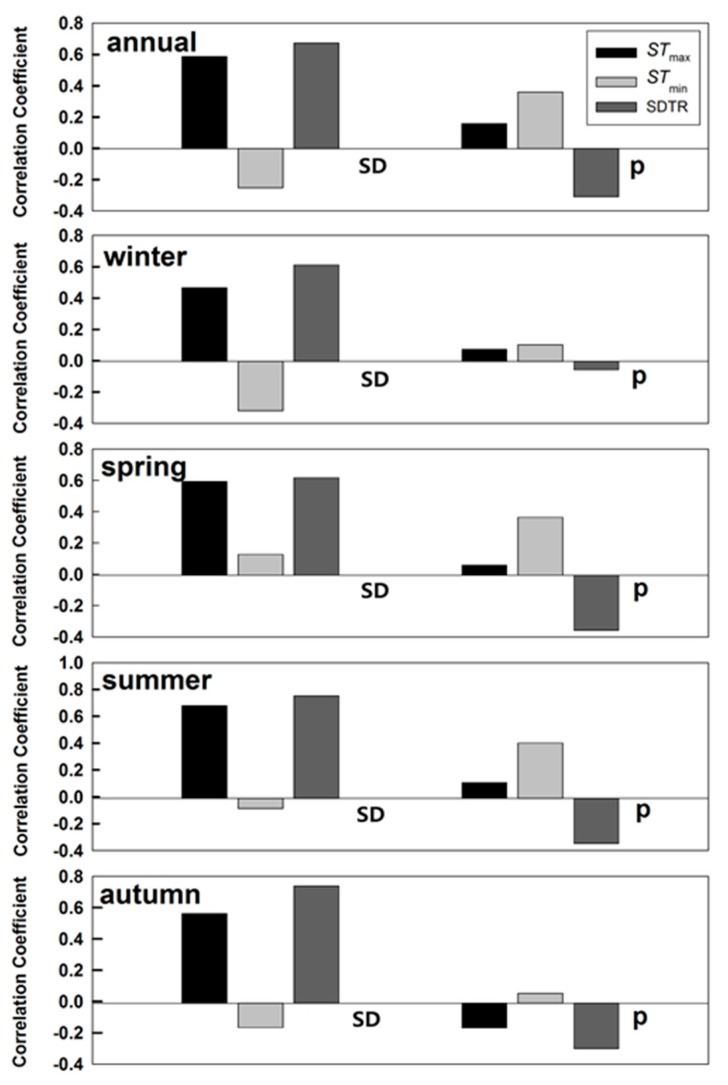
Partial correlation coefficients between $ST_{\max}$, $ST_{\min}$, SDTR, and sunshine duration (SD), precipitation (p) on annual and seasonal scales in China during 1962–2011. All the correlation coefficients were significant (*p* < 0.05).

Table 3 showed the correlation coefficients among indicators (SD, p, h, and V) for each season. SDTR had a strong positive correlation with SD and a strong negative correlation with precipitation (Table 3 and Figure 5). Thus, energy and water conditions control SDTR.

**Figure 6:**
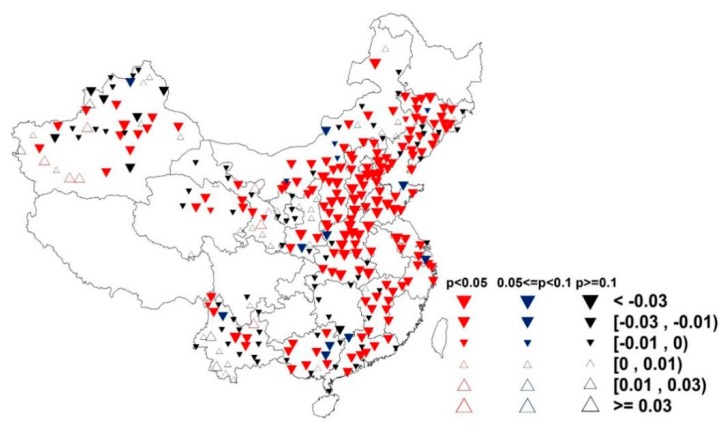
Spatial patterns of changes in sunshine duration (SD) during 1962–2011 (h/year) at 360 observational stations in China. The color of the circles indicates the significance level of change. Solid or hollow triangles indicate the magnitude of change. Maps in this figure were created by ArcGIS 9.3 (ESRI, 2008).

Sunshine duration is an important variable, which can be a proxy for SR (solar radiation) [24,25]. Reductions in SD have been attributed to the increasing atmosphere aerosols from industrial pollution, which reduces the amount of sunlight reaching the ground [26,27]. Furthermore, sunshine duration was closely related to changes in DTR [5,6]. For soil temperature (Table 3), SD had a high positive correlation (0.73) with SDTR (annual level). In day and night, respectively, SD was positively correlated (0.44) with $ST_{\max}$, while sunshine duration was directly related to downward shortwave radiation, which was initially received by the soil surface and subsequently spread to air. Consequently, a decreasing SD would reduce the downward shortwave radiation during the day, thereby limiting the increases in $ST_{\max}$ [6]. However, there is no explanation for the significant negative association (0.62) between SD and $ST_{\min}$, which is possibly due to the interaction between variables. Hence, we calculated the partial correlation coefficients of the variables with SDTR in order to eliminate the effects of any interaction between variables on SDTR (Figure 5). The results demonstrated no significant and obvious relationship between SD and $ST_{\min}$, indicating that $ST_{\max}$ was the primary factor behind the significant positive association between SD and SDTR. The SD was highly correlated with SDTR in each season. The magnitude of correlation coefficients follows the order of winter (0.78) > autumn (0.77) > spring (0.74) > summer (0.61). In addition, looking at the trends in SD changes over the same time period (Figure 6), we found that the areas of decreasing SD correspond to areas where SDTR had also decreased (Figure 3). Based on the strong positive correlation and similar spatial patterns, we concluded that the decreases in SD were largely contributed to the decreases in SDTR.

**Figure 7:**
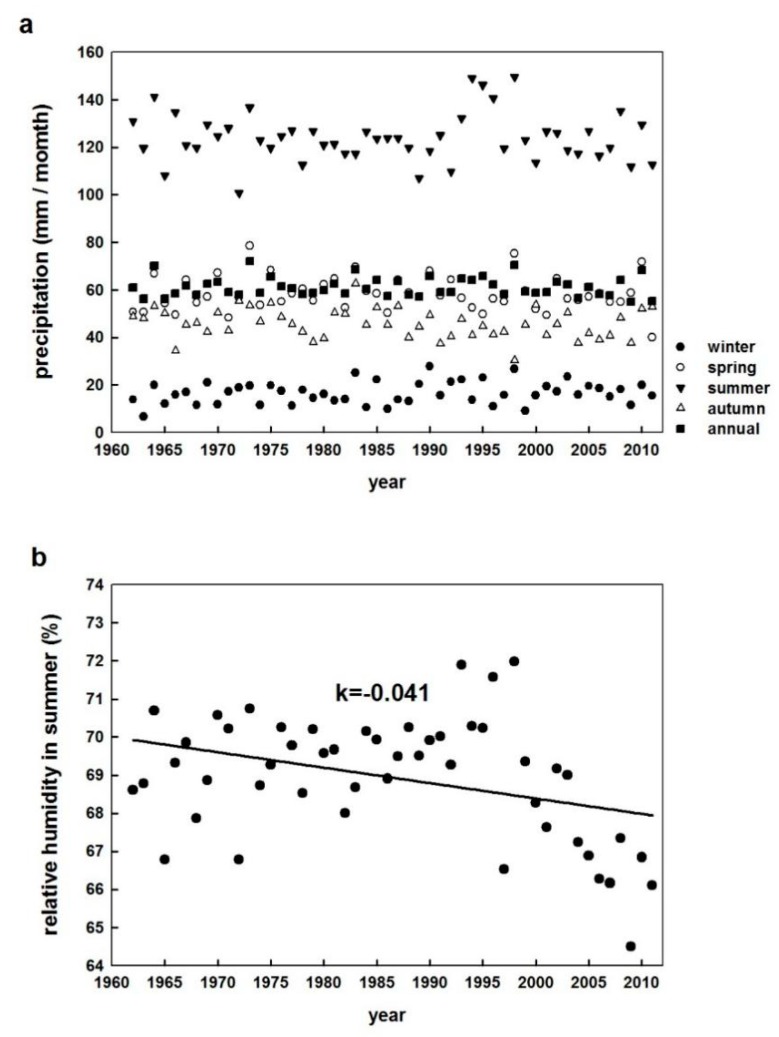
(**a**) Precipitation (p) on annual and seasonal scales in China during 1962–2011. The annual averaged values of 12 monthly accumulated precipitations were used in this graph. Linear trends of precipitation were not significant (*p* > 0.05). (**b**) Relative humidity in summer in China during 1962–2011. The seasonal averaged values (summer) of daily mean relative humidity were used in this graph. The solid line represents the linear trend and k represents the rate of change (%/year). r^2^ = 0.38, *p* < 0.05.

The variations in soil moisture alter the partitioning of sensible heat and latent heat loss from the surface and affect the atmospheric boundary-layer processes and regional circulation [28,29]. Therefore, other factors affecting SDTR may include precipitation and relative humidity, which would alter soil temperature by impacting heat transfer. Precipitation and SDTR had a negative relationship over the study period (correlation coefficient of –0.55; Table 2). Precipitation had different effects on soil temperature in the day compared to night. Precipitation had a strong negative effect on $ST_{\max}$ (–0.47 in annual level) but the correlation between the precipitation and $ST_{\min}$ was statistically non-significant on the annual and seasonal time scales, except during winter months. However, the changes in precipitation were not significant on an annual level and each seasonal time scale (Figure 7a and Shen, 2014). Therefore, we inferred that precipitation was not the main reason for SDTR changes in China. Relative humidity decreased during the study period (Figure 7b) but it was not correlated with SDTR. 

There are regional differences in climatic and landform conditions in China, as temperatures generally increase from north to south [30]. In contrast, precipitation and humidity decrease from coastal to inland areas [15]. Thus, this is a possible limitation for future studies analyzing seasonal changes in SDTR on such a large regional scale. 

### 3.4. Soil and Air Temperature Differential

The difference between soil and air temperature ($T_{D}$) is a key index of surface heating flux. The surface sensible heat flux is not only the primary energy source of the lower atmosphere but also is important in surface heat balance. In addition, crop growth is affected by soil and air temperature. Temperature is one of the most important factors to keep plants alive and functional [18,31]. Seed germination, seedling emergence, developmental and growth, root extension and growth, and fruit maturation are sensitive to air temperature and soil temperature changes. Furthermore, they are sensitive to the dynamic relation of air and soil temperatures.

**Figure 8:**
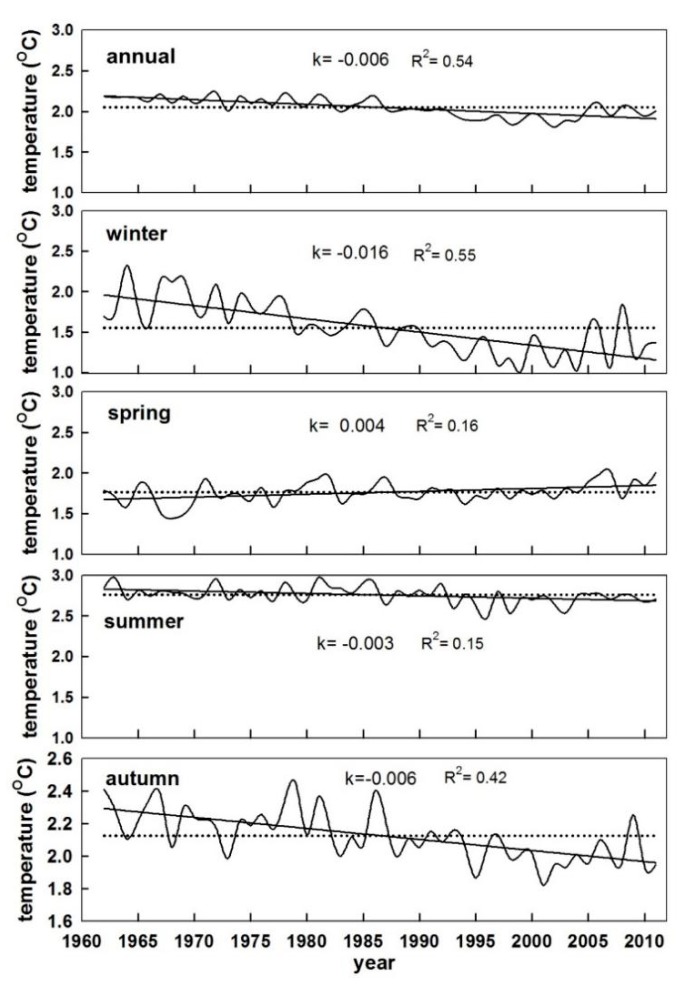
Linear trends from annual means of daily $T_{D}$ on annual and seasonal scales in China during 1962–2011. Dashed lines represent averages. K represents the slope (i.e., the rate of change (°C/year)); all the slopes of trends were significant (*p* < 0.05).

In the past 50 years, the average annual difference between the mean surface soil temperature and mean air temperature was 2.05 °C (Figure 8). Across seasons, $T_{D}$ was highest in summer (2.76 °C), followed by autumn (2.13 °C) and spring (1.76 °C). Furthermore, it was smallest in winter (1.55 °C). Across all stations, the average $T_{D}$ decreased by 0.3 °C over 50 years. The decrease in $T_{D}$ was highest in winter (0.8 °C), followed by that in summer (0.3 °C) and autumn (0.15 °C). The opposite trend was found in summer (increase of 0.2 °C).

**Figure 9:**
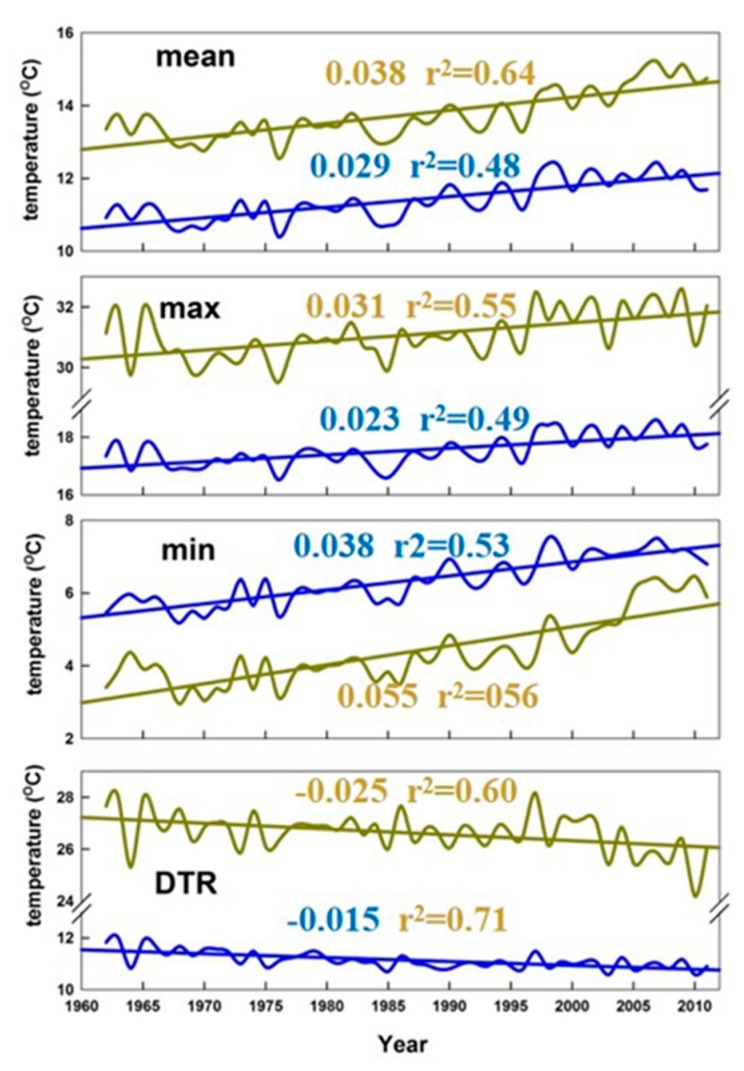
Time series and linear trends from annual means of $ST_{mean},ST_{\max},ST_{min},SDTR$ (yellow lines and numbers), $AT_{mean},AT_{\max},AT_{min},DTR$ (blue lines and numbers) for China during 1962–2011. Values along the trends lines are rate of change (°C/year), all the slopes of trends were significant (*p* < 0.05).

The difference between the soil temperature and air temperature is of great significance for plant growth and agricultural production practice, especially for sowing and germination processes. Looking at Figure 9, $ST_{\min}$ < $AT_{\min}$(i.e., soil temperatures were less than air temperatures at night). This meant when the air temperature was above 0 °C, the soil temperature was likely below 0 °C. The temperature conditions at this time may not be available for plant survival and, hence, agricultural practice plans based on air temperature alone are severely limited. In particular, for perennial species, their resistance to overwintering is a serious challenge. Moreover, as the process of root growth and extension is not visible in soil, it is difficult to detect the problem and take action. Over the past half century, according to Figure 9, the difference between the $ST_{\min}$ and $AT_{\min}$ reduces at night. This would facilitate the wintering of perennials in areas near the zero-contour line ($AT_{\min}$).

## 4. Conclusions

This paper revealed asymmetric soil warming in two aspects. Firstly, there was asymmetry between day and night in terms of increases in soil temperature. Secondly, there was asymmetry between the soil and air in the temperature increase. 

For China, the average SDTR, which is the soil temperature range between day and night, decreased at an average of 0.025 °C/year during the study period due to a slight increase in $ST_{\max}$ and a substantial increase in $ST_{\min}$ (0.031 and 0.055 °C/year, respectively) in all seasons at most stations. The annual decrease in SDTR was mainly attributed to a decrease in winter (–0.08 °C/year). The solar duration was positively and strongly related to SDTR, whereas precipitation was negatively associated with SDTR. Based on the significant reductions in SD change and similar spatial patterns between SD and SDTR, we concluded that the decreasing SD was an important cause of the decrease in SDTR. In general, soil temperature changes were consistent with those of air temperature, although the magnitude of their asymmetry affected crop growth. The differences between soil and air temperature were highest in summer (2.76 °C) and smallest in winter (1.55 °C). On average, $T_{D}$ decreased by 0.3 °C over the entire period, which was mainly attributed to a substantial change in winter (0.8 °C over 50 years). The asymmetry between soil temperature and air temperature meant agricultural practice plans based on air temperature alone may be severely limited. The difference between soil temperature and air temperature reduces at night. This would facilitate the wintering of perennials in areas near the zero-contour line. These results about asymmetric soil warming are important because it highlights the significance of using soil temperature (versus air temperature) for a reliable estimation of the responses and feedback of ecological soil processes to climate change and agricultural production.

## Figures and Tables

**Table 1:** Variables used in this paper and their descriptions.

Variable	Description
$ST_{mean}$	daily mean surface soil temperature
$ST_{\max}$	daily maximum surface soil temperature
$ST_{\min}$	daily minimum surface soil temperature
SDTR	$ST_{\max}$–$ST_{\min}$, diurnal range of surface soil temperature
$AT_{\mathrm{mean}}$	daily mean air surface temperature
$AT_{\max}$	daily maximum air surface temperature
$AT_{\min}$	daily minimum air surface temperature
DTR	$AT_{\max}$–$AT_{\min}$, diurnal range of air temperature
SD	daily sunshine duration
h	daily mean relative humidity
V	daily mean surface wind speed
p	daily accumulation of precipitation
$T_{D}$	$ST_{mean}-AT_{mean}$

**Table 2:** National trends of $ST_{\max}$, $ST_{\min}$, and SDTR on annual and seasonal scales in China during 1962–2011.

Season	*ST*_max_ (°C/year)	*ST*_min_ (°C/year)	SDTR (°C/year)
Annual	0.031 *	0.055 *	–0.025 *
Winter (DJF)	0.017	0.097 *	–0.080 *
Spring (MAM)	0.048 *	0.045 *	0.003
Summer (JJA)	0.023 *	0.034 *	–0.011
Autumn (SON)	0.033 *	0.045 *	–0.012

* *p* < 0.05.

**Table 3:** Correlation coefficients between soil temperature variables ($ST_{\max}$, $ST_{\min}$, and SDTR) and other climatological variables including DTR, sunshine duration (SD), precipitation (p), relative humidity (h), and surface wind speed (V) on annual and seasonal scales in China during 1962–2011.

Variable	$ST_{\max}$	$ST_{\min}$	SDTR
Annual			
SD	0.44 *	–0.62 *	0.73 *
p	–0.47 *	0.06	–0.55 *
h	–0.12	–0.42	–0.21
V	–0.59 *	–0.74 *	0.31 *
Winter			
SD	0.46 *	–0.50 *	0.78 *
p	–0.44 *	0.28 *	–0.57 *
h	–0.23 *	–0.01	–0.15
V	–0.20	–0.62 *	0.43 *
Spring			
SD	0.40 *	–0.30 *	0.74 *
p	–0.41 *	0.20	–0.68 *
h	–0.30	–0.14	–0.13 *
V	–0.57 *	–0.72 *	–0.12
Summer			
SD	0.12 *	–0.55 *	0.61 *
p	–0.41 *	0.14	–0.61 *
h	–0.37 *	–0.39 *	–0.06
V	–0.36 *	–0.72 *	0.18
Autumn			
SD	0.20 *	–0.53 *	0.77 *
p	–0.59 *	0.01	–0.64 *
h	–0.28 *	–0.07	–0.34 *
V	–0.59 *	–0.66 *	0.07

* *p* < 0.05.
